# Alexithymia Predicts Attrition and Outcome in Weight-Loss Obesity Treatment

**DOI:** 10.3389/fpsyg.2018.02432

**Published:** 2018-12-04

**Authors:** Mario Altamura, Piero Porcelli, Beth Fairfield, Stefania Malerba, Raffaella Carnevale, Angela Balzotti, Giuseppe Rossi, Gianluigi Vendemiale, Antonello Bellomo

**Affiliations:** ^1^Department of Clinical and Experimental Medicine, University of Foggia, Foggia, Italy; ^2^Department of Psychological, Health and Territorial Sciences, D’Annunzio University of Chieti–Pescara, Chieti, Italy; ^3^CeSI-Met, D’Annunzio University of Chieti–Pescara, Chieti, Italy

**Keywords:** diagnostic criteria for psychosomatic research, predictive factors, obesity, clinical intervention, dropout, alexithymia

## Abstract

Obesity is a psychosomatic condition characterized by a complex interaction of biological and psychological factors and a large body of research has aimed to identify variables limiting efficacy and determining high attrition rates in weight loss programs. In this study, we used the Diagnostic Criteria for Psychosomatic Research (DCPR), designed to broaden the clinician’s perspective on patients’ problems by providing additional clinical information not found in the more traditional psychiatric classification, to predict psychosomatic variables that may limit efficacy and determine attrition in clinical interventions with people with obesity. We evaluated 82 consecutive participants with obesity at baseline for psychopathology, psychosomatic correlates, psychological distress, and eating-related symptoms before entering a weight loss program. Regression models were used to assess attrition and outcome at a 6-month follow-up and per-protocol and intention-to-treat analyses were performed. DPCR alexithymia significantly predicted attrition (OR = 6.9), and unsuccessful weight-loss (OR = 11.3). These findings suggest that the identification of psychosomatic factors, in addition to psychological and psychopathological factors, may predict adherence to weight-loss programs.

## Introduction

Obesity is a multifactorial condition, characterized by complex interactions between biological and psychological factors, that results in the accumulation of adipose (fat) tissue due to increased caloric intake and/or decreased expenditure of calories (Karasu, 2012). Moreover, obesity has become a major health concern in developing countries and is considered a risk factor for many non-communicable diseases including type 2 diabetes, cardiovascular disease (Ortega et al., 2016), musculoskeletal problems and many cancers (Stone et al., 2018). Obesity also affects self-esteem and is often associated with anxiety (Gariepy et al., 2010), social isolation and mood problems (Naper et al., 2017). Thus, as the prevalence and consequences of obesity rise, it is essential for clinicians and researchers to investigate treatment options and outcomes for patients with obesity.

Generally, behavior modification is the first recommended step in obesity management. Key features include self-monitoring, goal setting, nutrition, exercise, stimulus control, problem solving, cognitive restructuring, and relapse prevention (Poston and Foreyt, 2000; Reed, 2014). In particular, lifestyle modifications, including dieting and exercise, are essential for any treatment of obesity. Nonetheless, behavioral modification programs generally show attrition rates that range from 10 to 80% within a few days of initiating treatment (Farley et al., 2003), only small changes in targeted behaviors (van Sluijs et al., 2004; Lakerveld et al., 2013) and relatively little weight loss is maintained across time (Wing and Hill, 2001).

Identifying factors that determine attrition and weight outcome are, therefore, critical for weight-loss interventions in people with obesity. In this regard, however, literature reports very mixed results and factors investigated are inconsistent. Some studies have found multiple pre-treatment variables (e.g., demographic, weight-related and dietary habits) to be associated with attrition and successful weight loss (for reviews see, e.g., Teixeira et al., 2002, 2005; Dalle Grave et al., 2006). Others show that psychological and psychosocial variables determine attrition and successful weight-loss outcomes (Dalle Grave et al., 2005, 2009; Grossi et al., 2006; Michelini et al., 2014). For example, a previous review of psychological factors and attrition in weight-loss treatments found lack of motivation, unrealistic weight loss expectations, lower level of physical, mental and weight-related quality of life and high levels of stress to be predictive (Dalle Grave et al., 2006). Other studies report associations with depression (Inelmen et al., 2005; Fabricatore et al., 2009), high levels of anger and hostility (Colombo et al., 2014), binge eating (Teixeira et al., 2004), alterations of body image (Traverso et al., 2000; Teixeira et al., 2004), poor interaction with therapists (Grossi et al., 2006), restricted and disinhibited eating, lower self-efficacy and self-motivation, personality traits, lower self-esteem, greater body dissatisfaction and internal locus of control (for overall reviews see Teixeira et al., 2005; Moroshko et al., 2011). Finally, other studies have shown associations with factors underlying constructs in psychosomatic literature such as difficulty expressing subjective emotional feelings and coping with emotions (De Chouly De Lenclave et al., 2001; Fassino et al., 2003; Pinaquy et al., 2003; Elfhag and Rössner, 2010; Da Ros et al., 2011; Pinna et al., 2014; Fernandes et al., 2018), personality traits (Sullivan et al., 2007; De Panfilis et al., 2008; Dalle Grave et al., 2018), and impulsiveness management (Van Hout et al., 2004; Leombruni et al., 2014; VanderBroek-Stice et al., 2017).

Few studies, however, have investigated the influence of these last factors on attrition and efficacy of weight-loss programs and those that did reported remarkably divergent findings. Sullivan et al. (2007) reported that adults with obesity scored higher for novelty seeking compared to lean controls. Moreover, these subjects were less successful in achieving weight loss compared to people with obesity who had a lower novelty-seeking trait. De Panfilis et al. (2007) found that individuals with obesity with lower narcissist traits showed poorer response to weight-loss interventions and Munro et al. (2011) reported neuroticism to be associated with successful weight loss. De Panfilis et al. (2008) found that low scores in reward dependence were predictive of attrition. In addition, impulsiveness, a multidimensional personality trait that can lead to uncontrolled eating, also seems to contribute to obesity development (Sutin et al., 2011; Giel et al., 2017; Ural et al., 2017). Indeed, studies investigating various aspects of impulsivity, including cognitive restraint, disinhibition (i.e., disinhibited eating behavior), and emotional eating (i.e., susceptibility to overeat in relation to emotional states), reported significant associations between impulsiveness and obesity (Hays and Roberts, 2008; Mobbs et al., 2010; VanderBroek-Stice et al., 2017). In particular, studies examining correlations of rigid and flexible restraint and treatment outcomes reported that an increase in cognitive restraint was associated with successful weight reduction and weight maintenance (Cuntz et al., 2001; Lejeune et al., 2003). Other studies failed to find any relationship between personality traits and outcomes in weight-loss programs (Teixeira et al., 2005).

In sum, results regarding attrition and outcomes in weight-loss interventions are controversial and inconsistent. Moreover, cross study comparisons on attrition and efficacy are complicated due to the multifactorial nature of predictive factors, the complex overlap of the psychological constructs investigated (Lazzeretti et al., 2015), and the variety of tools used to assess outcomes and no reliable factors have yet been established.

Here, we aimed to other identify factors that may influence attrition and outcome in patients with obesity through the Diagnostic Criteria for Psychosomatic Research (DCPR). The DCPR is a diagnostic and conceptual framework (Fava et al., 1995) that provides clinicians with a comprehensive set of diagnostic criteria specifically developed to translate psychosocial variables into categorical criteria with prognostic and therapeutic value (Porcelli and Guidi, 2015) and has been shown to detect subthreshold disorders and clinically relevant psychosocial distress even in the absence of any DSM-based psychiatric diagnosis (Bellomo et al., 2007; Guidi et al., 2013). In particular, the DCPR set is composed of 12 psychosomatic syndromes. Eight concern the main manifestations of abnormal illness behavior (somatization, hypochondriacal fears and beliefs, and illness denial) and four psychological factors affecting medical conditions (alexithymia, type A behavior, demoralization, and irritable mood). The 12 syndromes are assessed through a structured interview consisting of 58 items with a dichotomous “yes/no” response format and are useful for predicting treatment outcomes and psychosocial functioning in different medical settings (Porcelli et al., 2003; Porcelli and Rafanelli, 2010; Altamura et al., 2015a).

Venditti et al. (2013) explored the relationship of DCPR diagnoses with psychological well-being (PWB) and psychiatric disorders in patients with morbid obesity and normal weight controls and found that patients with a higher number of DCPR syndromes, showed lower psychological well-being (i.e., Autonomy and Self-Acceptance) as assessed by the PWB questionnaire. In addition, psychosomatic syndromes such as Health Anxiety and Demoralization, were more frequent in patients with obesity than in normal weight controls suggesting that feelings of demoralization and health-related concerns in the obese might hamper efforts to deal with excess weight, thus suggesting the clinical relevance of psychosomatic factors in contributing to and predicting poor compliance and outcome in weight-loss interventions. We aimed to replicate these findings and, by adopting a longitudinal design, to evaluate whether pre-treatment psychosomatic syndromes can identify adults with obesity at risk for attrition and/or poor outcome in weight-loss interventions.

## Materials and Methods

Ninety-five participants with obesity were recruited from the Clinical Nutrition Unit at the University Hospital of Foggia (Italy) from June 2017 to February 2018 participated in the study. Exclusion criteria included a Body Mass Index ranging below 30 or above 62, severe and/or chronic medical conditions (e.g., diabetes mellitus, thyroid illnesses, and heart disease), acute psychiatric disorders, and substance abuse within 6 months, and/or current use of psychotropic medication, as revealed through a baseline medical evaluation. On the first visit, all participants completed a self-report questionnaire on socio-demographic characteristics and medical history and completed a baseline medical evaluation to detect unstable medical conditions that could influence eligibility, associated disease risk and the degree of obesity. Thirteen participants, met exclusion criteria, thus leaving 82 participants for analyses. In particular, six for Type II diabetes, three for cardiovascular problems, two for psychotherapeutic drugs, one for osteoarthritis, and one for hyperuricemia. The baseline medical evaluation included an ECG, a physical examination, and laboratory tests including serum thyroid hormones level, glucose serum level, serum total cholesterol, high- and low-density lipoprotein (HDL, LDL) cholesterol, and triglyceride. BMI (Kg/m^2^) was measured with a DETECTO physician scale with a precision of 0.2 Kg. Height was measured with a stadiometer with a precision of 0.5 cm (Altamura et al., 2015b). After successful completion of the baseline medical evaluation, clinicians prescribed a personalized hypocaloric diet. Participants received nutritional counseling every 4 weeks for the duration of the weight-loss intervention and the 6-month follow-up period. In particular, the clinical trial lasted 12 months; in the present study, we examine the attrition trend during the first 6 months. Standard care consisted in a low calorie diet (1200–1350 kcal/d) with 15–20% of the energy intake from protein, 25–30% from lipid and 50–55% from carbohydrates in accordance with the Italian and European Guidelines (Michelini et al., 2014). The program consisted in an initial clinical evaluation at the outpatient clinic, followed by control visits every 4 weeks for a total of six visits in the first 6 months (considering baseline as the initial visit). On the first visit, the patient met both the physician and dietitian for the physical examination and for dietary counseling. On the follow-up visits, the physician and dietitian assisted the patient in the monitoring of weight and addressed themes such as nutrition education in order to promote dietary changes. On all visits, every patient received similar clinical evaluation and dietary counseling.

At baseline, participants completed the International Neuropsychiatric Interview (MINI) to assess psychiatric morbidity (Sheehan et al., 1998) and the Structured Interview for DCPR (Porcelli and Guidi, 2015) to assess psychosomatic syndromes. The Symptom Checklist-90R (SCL-90-R) was used to assess psychopathological distress. This checklist consists of 90 items with Likert response scores resulting in nine syndromes (depression, anxiety, phobic anxiety, hostility, obsessive-compulsive, interpersonal sensitivity, somatization, paranoid ideation, and psychoticism dimensions). The SCL-90-R global severity index (GSI) measures the extent or depth of the individual’s psychiatric disturbances (Derogatis and Cleary, 1977). The Eating Attitude Test (EAT-26), a 26-item measure, was used to assess a broad range of disordered eating behaviors including dieting and food preoccupation (Garner et al., 1982). The 26 items are classified under the following three separate subscales: (1) “Dieting,” (2) “Food Preoccupation and Bulimia,” and (3) “Oral control” (Orbitello et al., 2006). The responses on the 26 items are summed at the end and a total score, ranging from 0 (minimum) to 78 (maximum), is extracted. Scores >20 indicate a tendency to develop eating disorders. Further, responses in each subscale are extracted from the sum of its respective items.

Two outcome measures were considered, attrition rate and weight-loss success. Weight changes were analyzed for completers only (per-protocol approach) and following an intent-to-treat approach known as the baseline observation carried forward (BOFC) method to include baseline data for all participants (Ware, 2003). Successful participants were those who lost 5% or more of their initial body weight (T0) after 6 months (T1). Non-successful participants were defined as those showing no weight loss or weight gain (i.e., weight change ≥ 0.5 kg) at 6 months. Dropouts were considered unsuccessful weight-loss patients in line with the assumption that those who dropped out returned to their baseline weight within 6 months. The baseline observation carried forward procedure (BOCF) was used to define success categories, thus classifying all dropouts as non-successful (no change from baseline). All other participants, including those showing weight changes smaller than 5% were excluded from analyses when comparing the two success categories. This procedure was chosen to identify two clearly distinct levels of success and minimize misclassification caused by using a single cutoff separating the two success levels (Teixeira et al., 2004).

The protocol was approved by the local Ethic Committee: Azienda Ospedaliero-Universitaria, “Ospedali Riuniti” di Foggia. All participants gave written informed consent in accordance with the Declaration of Helsinki.

### Statistical Analysis

Dropouts for attrition were considered patients who missed two consecutive visits (Michelini et al., 2014). Improved outcome was considered as ≥5% weight loss after 6 months (T1) compared to initial body weight (T0) (Jiandani et al., 2016). Parametric (χ^2^) and non-parametric tests (Fisher’s test) were used to compare baseline data of completers vs. non-completers and successful vs. unsuccessful weight-loss participants. Multivariate logistic regression models were used to establish predictors of dependent variables (attrition rate and weight loss success). The DCPR syndromes diagnosed in more than 10% of participants, DCPR severity (individuals with more than one diagnosis, DCPR > 1), baseline MINI diagnoses, and psychological distress (SCL-90-R-GSI total score; EAT-26) were considered as predictors. Relationships between each predictor and outcome were initially tested using univariate logistic regressions. Predictors found to be significantly (*p* < 0.05) related to outcomes in the univariate analyses were simultaneously entered into a multivariate model to predict outcomes. Multicollinearity among predictors was assessed using a conventional tolerance threshold of <0.20. Tolerance values less than 0.20 indicate presence of collinearity (Fabricatore et al., 2009). The level of statistical significance was set at *p* < 0.05.

## Results

Included participants and excluded patients were not different for age, gender, education, weight, and BMI. Demographic, waist circumference and baseline psychosocial characteristics of the entire study sample and differences between completers and non-completers are presented in Table 1.

**Table 1:** Description of the study sample.

Characteristics	Total (*n* = 82)*n* (%) mean ± sd	Completers (*n* = 36)*n* (%) mean ± sd	Noncompleters (*n* = 46)*n* (%) mean ± sd	*P*-values (χ^2^,Fisher’s test)
Age (yrs)	46.9 ± 11.7	47.0 ± 12.1	46.8 ± 10.6	0.93
Females (%)	67 (81.7)	29 (35.3)	38 (46.3)	0.53
Education (yrs)	9.9 (3.2)	9.8 ± 2.9	9.9 ± 3.4	0.92
BMI (Kg/m^2^)	39.4 ± 6.9	39.4 ± 5.8	39.3 ± 7.6	0.92
Weight (Kg)	100.5 ± 21.7	98.9 ± 23.9	101.8 ± 20.1	0.56
Medical comorbidities (hypertension; hyperlipidemia)	25 (30.4)	10 (12.1)	15 (18.2)	0.26
**DCPR diagnoses**				
Alexithymia	20 (24.3)	2 (2.4)	18 (21.9)	0.0001
Irritable Mood	15 (18.2)	3 (3.6)	12 (14.6)	0.009
Demoralization	13 (15.8)	6 (7.3)	7 (8.5)	0.55
Illness Denial	12 (14.6)	6 (7.3)	6 (7.3)	0.46
Type A Behavior	12 (14.6)	3 (3.6)	9 (10.9)	0.04
DCPR > 1	27 (32.9)	4 (4.8)	23 (28.0)	0.0001
**MINI diagnoses**				
Depressive disorders	18 (21.9)	10 (12.1)	8 (9.7)	0.33
Anxiety disorders	13 (15.8)	5 (6.0)	8 (9.7)	0.47
SCL-90-R-GSI total score	0.76 ± 0.6	0.55 ± 0.4	0.93 ± 0.7	0.004
EAT-26 (Total score)	10.7 ± 3.3	10.3 ± 3.5	11.1 ± 3.2	0.84
EAT-26 (Total score > 20)	2 (2.4)	1 (1.2)	1 (1.2)	0.19
Dieting	4.1 ± 1.3	3.9 ± 0.5	4.3 ± 1.0	0.21
Food Preoccupation	5.0 ± 0.8	4.9 ± 0.7	5.2 ± 0.9	0.26
Oral Control	1.6 ± 1.0	1.6 ± 1.1	1.6 ± 0.9	0.48


*Results are reported only for DCPR and psychiatric diagnoses diagnosed in more than 10% of participants. DCPR, Diagnostic Criteria for Psychosomatic Research; MINI, Mini-International Neuropsychiatric Interview; SCL-90, Symptom Checklist-90; EAT-26, Eating Attitude Test-26.*

With regards to psychiatric diagnoses, thirty-three participants (40.2%) received a psychiatric diagnosis, the most frequent of which were depressive disorders and anxiety disorders. Only one patient had a diagnosis of Binge eating Disorder. Fifty-seven participants (69.5%) received a psychosomatic diagnosis. The DCPR diagnoses present in the sample included Alexithymia, Irritable Mood, Demoralization, Illness Denial, and Type A Behavior. Regarding, instead completers and non-completers, thirty-six patients with obesity (43.9%) completed the 6 month ambulatory intervention while 46 (56.1%) did not. Compared to completers, non-completers showed higher rates of Alexithymia, Irritable Mood, Type A Behavior, greater DCPR severity (DCPR > 1), and greater psychopathological severity (SCL-90-R-GSI total score). No significant differences were found for sociodemographic and illness-related variables. Surprisingly, psychiatric diagnoses were distributed in a similar manner between the two groups.

Table 2 shows odd ratios and 95% confidence intervals for univariate logistic regression models. Significant predictors of attrition from the univariate analysis (Alexithymia, Irritable Mood, DCPR > 1, EAT-26-Dieting and SCL-90-R-GSI total score) were simultaneously entered in the multivariate logistic regression model. An interesting result concerns the positive correlation between EAT-26-Dieting scores and attrition. This is in line with previous studies that reported a strong correlation between abnormal eating attitudes, as measured by the EAT-26, and psychopathological disorders in obese women (Altamura et al., 2015b).

**Table 2:** Results of univariate logistic regression analyses to predict attrition and weight loss success.

Independent variables	Drop-out			Weight loss		
	OR	95%CI	*P*-values	OR	95%CI	*P*-values
Sex	0.41	0.30–0.59	0.51	0.11	0.8-1.2	0.73
Age	0.11	0.01–0.12	0.73	0.63	0.02-0.05	0.39
Education	0.06	0.04–0.05	0.79	0.04	0.13-0.16	0.83
Alexithymia	9.96	1.52–36.1	0.003	12.8	1.53-108.3	0.01
Irritable Mood	6.01	1.21–29.5	0.02	2.72	0.73-18.7	0.11
Demoralization	0.33	0.08–1.23	0.81	0.42	0.09-2.05	0.29
IllnessDenial	0.75	0.21–2.60	0.64	1.51	0.28-8.76	0.52
Type A Behavior	4.72	0.94–23.7	0.06	6.31	0.73-55.3	0.09
DCPR > 1	5.01	1.72–14.5	0.004	3.62	1.11-11.5	0.03
MINI diagnoses	0.82	0.33–2.03	0.66	0.76	0.27-2.15	0.61
SCL-90-R-GSI total score	8.87	0.11–0.53	0.002	3.91	0.04-0.47	0.04
EAT-26 (Total score)	0.87	0.32–1.22	0.22	2.71	0.09-019	0.09
EAT-26 (Total score > 20)	0.61	0.16–2.22	0.44	0.46	0.11-1.83	0.26
EAT-26-Dieting	3.73	0.01–0.17	0.05	0.52	0.05-0.11	0.46
EAT-26-Food Preoccupation	2.40	0.01–0.14	0.12	2.08	0.01-0.11	0.14
EAT-26-Oral Control	1.28	0.01–0.04	0.25	0.38	0.04-0.08	0.53


*DCPR, Diagnostic Criteria for Psychosomatic Research; SCL-90, Symptom Checklist-90; MINI, Mini-International Neuropsychiatric Interview; EAT-26, Eating Attitude Test-26.*

As shown in Table 3, multivariate logistic regression showed that only alexithymia significantly predicted attrition. All predictors in the models showed tolerance values above 0.20, indicating no multicollinearity. Among those who completed the weight-loss intervention, twenty-four participants (66.7%) showed an equal to or greater than 5% weight loss with respect to their initial body weight. Twelve patients (33.3%) did not exhibit such an improvement. Mean weight change for the successful weight-loss group was -7.9 kg (-8.0% of initial weight). There were no significant baseline differences between the two groups. Although no variable predicted weight change when comparing completers only, in the intention-to treat analysis unsuccessful weight-loss participants (*N* = 46) showed higher rates of baseline alexithymia (*p* = 0.003), greater DCPR severity (*p* = 0.01), and greater psychopathological severity (*p* = 0.02) compared to successful weight-loss participants (*N* = 24). Significant predictors of weight loss from the univariate analysis (alexithymia, DCPR > 1, SCL-90-R-GSI total score) were simultaneously entered in the multivariable logistic regression model showing that only alexithymia significantly predicted unsuccessful weight loss (Table 4).

**Table 3:** Results of multivariate logistic regression analysis to predict attrition.

Independent variables	OR	95%CI	*P*-values
Alexihtymia	6.88	1.24-38.0	0.002
Irritable Mood	1.77	0.19-15.8	0.61
DCPR > 1	1.42	0.30-6.62	0.64
SCL-90-R-GSI total score	2.78	0.03-0.45	0.09
EAT-26-Dieting	1.16	0.20-0.70	0.27


*OR, Odds ratio; CI, Confidence Interval; DCPR, Diagnostic Criteria for Psychosomatic Research; SCL-90, Symptom Checklist-90.*

**Table 4:** Results of multivariate logistic regression analysis to predict weight loss.

Independent variables	OR	95%CI	*P*-values
Alexithymia	11.3	1.12-114.9	0.04
DCPR > 1	0.93	0.20-4.16	0.92
SCL-90-R-GSI total score	2.95	0.14-2.19	0.08


*OR, Odds ratio; CI, Confidence Interval; DCPR, Diagnostic Criteria for Psychosomatic Research; SCL-90, Symptom Checklist-90.*

## Discussion

In this study, we aimed to extend results by Venditti et al. (2013) that demonstrated the importance of DCPR diagnoses in characterizing the functioning and psychological response to medical illness in people with obesity. Our main results suggest that the severity of psychosomatic burden, including subjective psychological distress, and in particular alexithymia, may play a clinically significant role in predicting attrition in weight management programs.

The high prevalence of DCPR alexithymia, irritable mood, illness denial, type A behavior, and demoralization found in our sample compare favorably with results reported by other researchers who investigated the frequency of DCPR diagnoses in eating disorders suggesting that those psychosomatic diagnoses may be common syndromes in obesity and eating disorders (Fassino et al., 2007). Interestingly, even though DCPR diagnoses have shown clinical utility in designing intervention programs in medical settings (Porcelli and Guidi, 2015), to our knowledge no previous study has assessed the association of DCPR diagnoses with attrition and outcome, critical aspects of obesity programs.

Discriminating psychological factors that can lead to successful weight loss and-premature program termination are also crucial. Here, we found alexithymia to be the strongest independent predictor of both premature termination and unsuccessful weight loss. Alexithymia, or the reduced ability to identify, describe and distinguish between different feelings, is thought to reflect deficits in the cognitive processing and regulation of emotions that affect health perception through dysregulation of stress-related emotional arousal and low tolerance to distressing stimuli (Lumley et al., 2007). In addition, neuroimaging evidence shows that alexithymia is associated with reduced neural responses to emotional stimuli from the external environment and enhanced neural activity in somatosensory and sensorimotor regions (Moriguchi and Komaki, 2013).

Alexithymic personality traits are frequently observed in people with obesity and eating disorders (Pinaquy et al., 2003; Fassino et al., 2007; Westwood et al., 2017) and a recent study by Pinna et al. (2014) suggests that alexithymic traits are predictive of dropouts from treatment programs in patients with eating disorders. In our study, a significant rate of patients with obesity, without comorbid eating disorders, diagnosed with DCPR alexithymia, dropped-out suggesting that individuals with alexithymia may be more likely to discontinue treatment due to difficulties in building collaborative therapeutic relationships (Grabe et al., 2001; De Panfilis et al., 2008; Berrocal et al., 2009; Vanheule et al., 2011), limited adaptive coping in stressful situations (Pinaquy et al., 2003), associations with unhealthy eating behaviors (Wheeler et al., 2005; Lumley et al., 2007), and/or poor differentiation between somatic and psychological emotional arousal and between the sensations of hunger and satiety (Fassino et al., 2004). Indeed, it is possible that an inability to discriminate between affect and sensation and between the sensations of hunger and satiety could have a negative impact on adherence to dietetic recommendations.

The findings of the present study highlight the importance of identifying alexithymic patients prior to treatment initiation and during weight loss treatment to prevent possible dropouts and promote adherence to weight loss interventions. Indeed, psychological treatments aimed at modifying alexithymic traits, and specifically those targeting emotions, often result in a significant increase of efficacy of therapeutic interventions in individuals with eating disorders (Pinna et al., 2014). Therefore, it is conceivable that these psychological interventions might also improve treatment adherence in alexithymic individuals with obesity.

Concerning psychiatric comorbidity, the proportion of patients with anxiety and depressive disorders within our subjects were similar in both completers and non-completers. This result was surprising as higher rate of psychiatric comorbidity in non-completers than completers was expected (De Panfilis et al., 2008; Fabricatore et al., 2009). This may be due to the use of different diagnostic methods for assessing psychiatric disorders. For example, in line with our results, De Panfilis et al. (2007)reported that completers did not differed from non-completers in observed rates of baseline depressive and anxiety disorders by using structured clinical interview.

The results of our study should be considered in the light of several limitations. First, the small sample size limits the generalizability of our results. Second, although comparable to other studies in literature (Minniti et al., 2004; Colombo et al., 2014), the high attrition rates found in our study may be related to other potentially clinically relevant variables such as treatment-related factors, motivation, self-efficacy, and self-esteem. The lack of assessment of those variables increase the probability of type II error and cannot account for mediating or moderating factors. In addition, this study is based on a substantial percentage of individuals with class III obesity who volunteered for short-term weight loss treatments, thus limiting the generalizability to patients with more severe levels of obesity who are less likely to benefit from short-term treatment. Also, we found substantial differences in the weight loss outcome when predictors were investigated by per-protocol (use of completers only) or intent-to-treat strategy (use of pre-treatment data for drop-outs), so that caution is warranted when comparing our findings with those obtained with different statistical methods. Although similar results have been observed in a previous study that used an intention-to-treat analysis (Teixeira et al., 2004) and we used an accurate model with a parsimonious subset of the predictors, multiple comparisons may have produced spurious associations.

## Conclusion

In conclusion, with these limitations in mind, findings from the present study suggest that clinicians should pay careful attention to the psychosocial characteristics of patients with obesity who accept to undergo short-term weight loss programs. Individuals with obesity who show higher psychological distress, relevant psychosomatic factors such as irritable mood and, particularly, alexithymic difficulties in identifying and describing their feeling states, are less likely to complete the treatment program and obtain benefits from it. Further controlled studies on larger samples are necessary to confirm our findings in order to tailor interventions to individual characteristics leading to enhance treatment adherence in diverse subgroups. Moreover, identification of predictive factors for adherence and weight-loss outcome in people with obesity maybe a pivotal strategy for reducing attrition and improving weight management when designing intervention programs.

## Author Contributions

MA and PP conceived and designed the experiments. GR, SM, RC, and AB performed the experiments. MA and PP analyzed the data. MA, BF, and PP wrote the manuscript. MA, PP, GV, and AB discussed results and provided comments.

## Conflict of Interest Statement

The authors declare that the research was conducted in the absence of any commercial or financial relationships that could be construed as a potential conflict of interest.
